# Association between systemic immune-inflammation index and latent tuberculosis infection: a cross-sectional study

**DOI:** 10.3389/fmed.2025.1615302

**Published:** 2025-07-30

**Authors:** Ting Pang, Lei Wang, Jie Zhang, Shuhong Duan

**Affiliations:** Department of Infectious Diseases, Beijing Shijitan Hospital, Capital Medical University, Beijing, China

**Keywords:** systemic immune-inflammation index, SII, latent tuberculosis infection, LTBI, NHANES

## Abstract

**Background:**

The systemic immune-inflammation index (SII) has been associated with various diseases, but its relationship with latent tuberculosis infection (LTBI) remains unclear. This study aimed to evaluate the association between SII and LTBI in United States adults.

**Methods:**

Data were obtained from the National Health and Nutrition Examination Survey (NHANES) 1999–2000 and 2011–2012 cycles. LTBI was defined as a positive result on either the QuantiFERON^®^-TB Gold In-Tube (QFT-GIT) assay or the tuberculin skin test (TST). SII was calculated based on neutrophil, platelet, and lymphocyte counts. All analyses were performed using complex survey design and sampling weights. Multivariable logistic regression models were applied to evaluate the association between SII and LTBI. SII was also analyzed in quartiles to assess trends. Restricted cubic spline (RCS) was employed to explore the potential non-linear associations. Subgroup analyses were conducted to assess whether the association varied across demographic and clinical strata.

**Results:**

A total of 9,489 participants were included, among whom 1,068 were identified with LTBI. Multivariable logistic regression demonstrated that SII was inversely associated with LTBI. For each 100-unit increase in SII, the odds of LTBI decreased by 6% (adjusted OR = 0.94, 95% CI: 0.90–0.97). When analyzed by quartiles, participants in the highest quartile had significantly lower odds of LTBI compared to those in the lowest quartile (adjusted OR = 0.58, 95% CI: 0.41–0.81), with a significant trend across quartiles (*P* for trend = 0.003). RCS showed a linear relationship between SII/100 and LTBI (*P* for non-linearity >0.05). The results of further subgroups analysis were consistent, with a significant interaction observed only for HIV status (*P* for interaction = 0.034).

**Conclusion:**

SII was inversely associated with LTBI and may serve as a readily accessible marker for LTBI risk stratification. Given its non-specific nature, further longitudinal studies are needed to validate its clinical and public health utility.

## 1 Introduction

Tuberculosis (TB) is an infectious disease that is widespread worldwide, caused by the bacterium *Mycobacterium tuberculosis* (Mtb), and poses a significant risk to human health. The World Health Organization (WHO) estimates that around 10.6 million people were infected with TB across the globe in 2021, with approximately 1.6 million reported deaths (1). In efforts to prevent and control TB, latent tuberculosis infection (LTBI) is an essential aspect that should not be ignored (2). LTBI signifies the existence of a sustained immune response within the body after being infected with Mtb, despite the lack of clinical symptoms or imaging abnormalities (3). It is estimated that nearly 30% of individuals who come into contact with Mtb will develop LTBI, and among these, 5% to 10% may progress to active TB within a few years after the initial infection (4). Therefore, effective identification and management of LTBI are crucial for diminishing the transmission and incidence of TB (5).

In recent years, the importance of the Systemic Immune-Inflammation Index (SII) in diagnosing, prognostic assessment, and monitoring disease progression across various medical conditions has gained increasing recognition (6–8). The SII is a comprehensive tool used to evaluate inflammation, derived from standard peripheral blood test parameters. The formula for calculating SII is as follows: SII = (neutrophil count × platelet count)/lymphocyte count, a method initially introduced by Hu et al. in 2014 (9). This index integrates neutrophils, platelets, and lymphocytes, which are key cellular components of the immune response, and functions as a novel biomarker indicative of systemic immune and inflammatory status (10). Tuberculosis, an inflammatory disease caused by Mtb infection, is characterized by a complex immune response (11). Given this, the link between SII and tuberculosis has garnered attention. Yu et al. found that the SII was significantly elevated in patients with active pulmonary tuberculosis compared to those with non-tuberculous pulmonary diseases (12). However, no studies have yet investigated the specific relationship between SII and LTBI. Consequently, this study utilized the National Health and Nutrition Examination Survey (NHANES) database to explore the connection between SII and LTBI, providing valuable insights into the health status of United States individuals.

## 2 Materials and methods data source

### 2.1 Study design and participants

The National Health and Nutrition Examination Survey (NHANES) serves as a crucial, ongoing evaluation of the health and nutritional conditions of the United States population. Our analysis used publicly available cross-sectional NHANES data, with sampling weights applied to account for the complex survey design. For detailed information on the NHANES methodology and updates, visit http://www.cdc.gov/nchs/nhanes/index.htm (accessed on 15 April 2025). Before data collection commenced, the study protocols were reviewed and approved by the ethical review board of the National Center for Health Statistics, ensuring that all participants were thoroughly informed and provided written consent for participation. Our analysis included individuals aged 18 and older who completed an interview, excluding those with missing data regarding SII or LTBI status.

### 2.2 SII measurement

Blood samples were collected at the Mobile Examination Center (MEC), where complete blood count (CBC) tests, including lymphocyte, segmented neutrophil, and platelet counts, were conducted using the Beckman Coulter^®^ HMX on EDTA-anticoagulated blood. The counts for neutrophils, lymphocytes, and platelets were recorded as × 10^3^ cells/μL. SII was calculated by multiplying the platelet count by the neutrophil count divided by the lymphocyte count (9).

### 2.3 LTBI measurement

In this study, LTBI was defined as a positive result on either the QuantiFERON^®^-TB Gold In-Tube (QFT-GIT) assay or/and the tuberculin skin test (TST) (13, 14). Data from the 1999–2000 and 2011–2012 NHANES cycles were included, as TST was conducted in both cycles and QFT-GIT was available in the 2011–2012 cycle. The QFT-GIT is an *in vitro* diagnostic test that measures interferon-gamma (IFN-γ) release in response to TB-specific antigens (15). Whole blood was collected into Nil, TB Antigen, and Mitogen tubes, incubated at 37°C ± 1°C for 16–24 h, and IFN-γ concentrations were quantified by enzyme-linked immunosorbent assay (ELISA). A result was considered positive if the IFN-γ level in the TB Antigen tube exceeded the Nil by ≥ 0.35 IU/mL and was ≥ 25% higher than the Nil, with Nil values ≤ 8.0 IU/mL. Samples with low Mitogen response (< 0.5 IU/mL) and no antigen response were classified as indeterminate and excluded. Only individuals with valid (positive or negative) results were included in the analysis. TST was considered positive if the skin induration was ≥ 10 mm (16, 17).

### 2.4 Covariates

The study accounted for several potential confounding factors, drawing on prior research and clinical expertise (18, 19). These included age, sex, race/ethnicity, family poverty-to-income ratio (PIR), educational attainment, marital status, body mass index (BMI), diabetes, coronary heart disease, hypertension, smoking status, alcohol consumption, hemoglobin levels, and platelet count. Age was treated as a continuous variable, while sex was categorized as male or female. Race/ethnicity was grouped as non-Hispanic white, non-Hispanic black, Mexican American, or other (18). Family income was divided into three categories based on the PIR: low income (PIR ≤ 1.3), medium income (PIR = 1.3–3.5), and high income (PIR > 3.5) (19). Education was classified into three levels: less than 9 years, 9 to 12 years, and more than 12 years (18). Marital status was categorized as either married or living with a partner, or living alone (18). BMI was calculated using standard procedures based on weight and height. Smoking status was classified as never smoked, current smoker, or former smoker (19). Similarly, alcohol consumption was classified into non-drinkers and drinkers. The presence of previous diseases (hypertension, diabetes, and coronary heart disease) was determined based on participants’ responses to the questionnaire regarding whether a doctor had diagnosed them with these conditions. Human immunodeficiency virus (HIV) status was defined by HIV-1 antibody testing and hepatitis B viruses (HBV) status by the presence of HBsAg detected through immunometric immunoassay, with participants classified as HIV-positive or -negative and HBV-positive or -negative accordingly (20). The outcome was a lifetime history of hepatitis C viruses (HCV) infection, defined by the presence of anti-HCV (21).

### 2.5 Statistical analysis

All the analyses in this study were conducted by using complex sampling design and sample weights. Baseline characteristics of participants with and without LTBI were compared using survey-weighted analyses. Continuous variables were summarized as mean with standard errors (SE), while categorical variables were reported as weighted proportions. Group comparisons were conducted using the Student’s *t*-test or Mann–Whitney U test for continuous variables and the chi-square test for categorical variables.

To examine the relationship between SII and LTBI, sample-weighted multivariable logistic regression analyses were conducted to estimate odds ratios (ORs) and 95% confidence intervals (CIs). Three progressively adjusted models were created to control for potential confounders. Model 1 was unadjusted, while Model 2 adjusted for key sociodemographic variables, including age, sex, race/ethnicity, family income, education level, marital status. Model 3 was fully adjusted, further incorporating BMI, diabetes, hypertension, coronary heart disease, smoking status, alcohol use, HIV, HBV, and HCV infection status. To confirm the association between SII and LTBI when treated as a continuous variable, SII was divided into quartiles and a trend test was conducted. Additionally, sample-weighted restricted cubic spline (RCS) regression was performed with four knots examine the dose-response relationship between SII and LTBI status. Subgroup analyses were conducted by sex, age, BMI, race/ethnicity, diabetes, smoking status, alcohol use, and HIV status. Within each subgroup, survey-weighted logistic regression models were applied using the fully adjusted model, excluding the stratification variable. To address missing covariate data, a multivariate single imputation approach was implemented using an iterative algorithm, with Bayesian Ridge regression as the estimator for successive imputations (22).

All statistical analyses were performed using R Statistical Software (Version 4.2.2; The R Foundation, Vienna, Austria^1^) and the Free Statistics software (Version 2.1.1; Beijing Free Clinical Medical Technology Co., Ltd., Beijing, China) (23). A two-tailed *p*-value < 0.05 was considered statistically significant. This cross-sectional study adhered to the STROBE (Strengthening the Reporting of Observational Studies in Epidemiology) guidelines.

## 3 Results

### 3.1 Baseline characteristics

**FIGURE 1:**
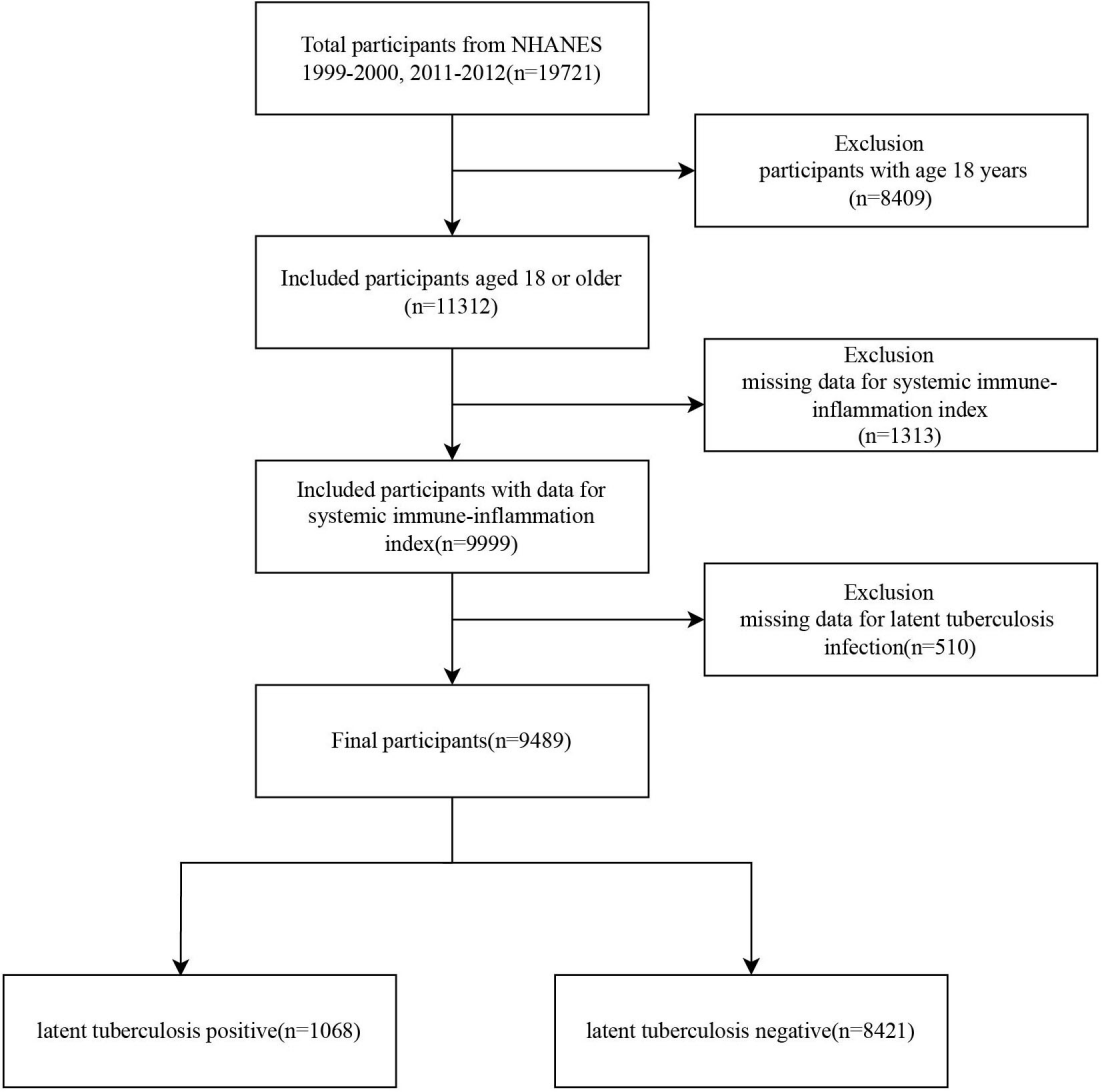
Flowchart of the study cohort. NHANES, National Health and Nutrition Examination Survey.

A total of 19,721 participants were initially selected from the NHANES 1999–2000 and 2011–2012 cycles. After excluding 8,409 individuals under 18 years of age, 11,312 participants remained. Of these, 1,313 were excluded due to missing data on the SII, and an additional 510 were excluded due to incomplete information on LTBI. Ultimately, 9,489 eligible participants were included in the final analysis, comprising 1,068 individuals with LTBI and 8,421 without LTBI (Figure 1).

Table 1 presents the weighted characteristics of the study population. The cohort consisted of 48.3% males, with a mean age of 45.4 years. The LTBI group was older, with an average age of 49.5 years, and had lower income and educational levels. Additionally, this group exhibited increased rates of hypertension and diabetes compared to the non-LTBI group. The LTBI group also showed lower neutrophil counts, platelet counts, and SII values than the non-LTBI group.

**TABLE 1:** Baseline characteristics of participants.

Variables	Total (unweighted *n* = 9489)	Non-LTBI (unweighted *n* = 8421)	LTBI (unweighted *n* = 1068)	p
Weighted, n	197,603,353	184,207,148	13,396,205	
Sex, (%)				**<0.001**
Male	48.25	47.63	56.81	
Female	51.75	52.37	43.19	
Age, years	45.40 (0.55)	45.1 (0.58)	49.5 (0.83)	**<0.001**
Race/ethnicity, (%)				**<0.001**
Non-Hispanic White	68.55	71.27	31.14	
Non-Hispanic Black	10.81	10.39	16.60	
Mexican American	7.24	6.48	17.68	
Other	13.41	11.87	34.57	
PIR, (%)				**<0.001**
low income	25.24	24.46	36.42	
medium income	34.37	34.24	36.19	
high income	40.39	41.30	27.39	
Education level years, (%)				**<0.001**
<9	6.39	5.54	17.80	
9–12	36.12	35.80	40.35	
>12	57.50	58.66	41.85	
Marital status, (%)				0.38
Living with partner	61.23	61.06	63.63	
Living alone	38.77	38.94	36.37	
BMI, kg/m^2^	28.30 (0.15)	28.30 (0.15)	28.25 (0.33)	0.98
Diabetes, (%)				**<0.001**
No	92.44	92.79	87.61	
Yes	7.56	7.21	12.39	
Coronary heart disease, (%)				0.82
No	97.00	96.98	97.16	
Yes	3.00	3.02	2.84	
Hypertension, (%)				**0.003**
No	77.54	77.95	71.82	
Yes	22.46	22.05	28.18	
Smoking status, (%)				0.067
Never	53.54	54.02	47.10	
Former	24.62	24.36	28.06	
Current	21.84	21.62	24.85	
Alcohol use, (%)				**0.005**
No	23.78	23.39	29.36	
Yes	76.22	76.61	70.64	
HIV, (%)				0.32
negative	99.55	99.53	99.77	
positive	0.45	0.47	0.23	
HBV, (%)				**0.001**
negative	99.69	99.74	99.00	
positive	0.31	0.26	1.00	
HCV, (%)				0.17
negative	98.35	98.40	97.66	
positive	1.65	1.60	2.34	
Lymphocyte (10^3^ cells/ul)	2.07 (0.02)	2.06 (0.02)	2.18 (0.04)	**0.004**
Neutrophil (10^3^ cells/ul)	4.28 (0.05)	4.30 (0.05)	4.06 (0.07)	**0.014**
Platelet (10^3^ cells/ul)	248.48 (1.38)	248.70 (1.43)	245.57 (2.63)	**0.018**
SII	561.80 (7.34)	566.35 (7.58)	499.23 (10.45)	**<0.001**

Mean (SE) for continuous variables, (%) for categorical variables. PIR, poverty income ratio; BMI, body mass index; SII, systemic immune-inflammation index; LTBI, latent tuberculosis infection; HIV, human immunodeficiency virus; HBV, hepatitis B virus; HCV, hepatitis C virus. Bold values indicate *p*-value < 0.05.

### 3.2 Association between SII and LTBI

**FIGURE 2:**
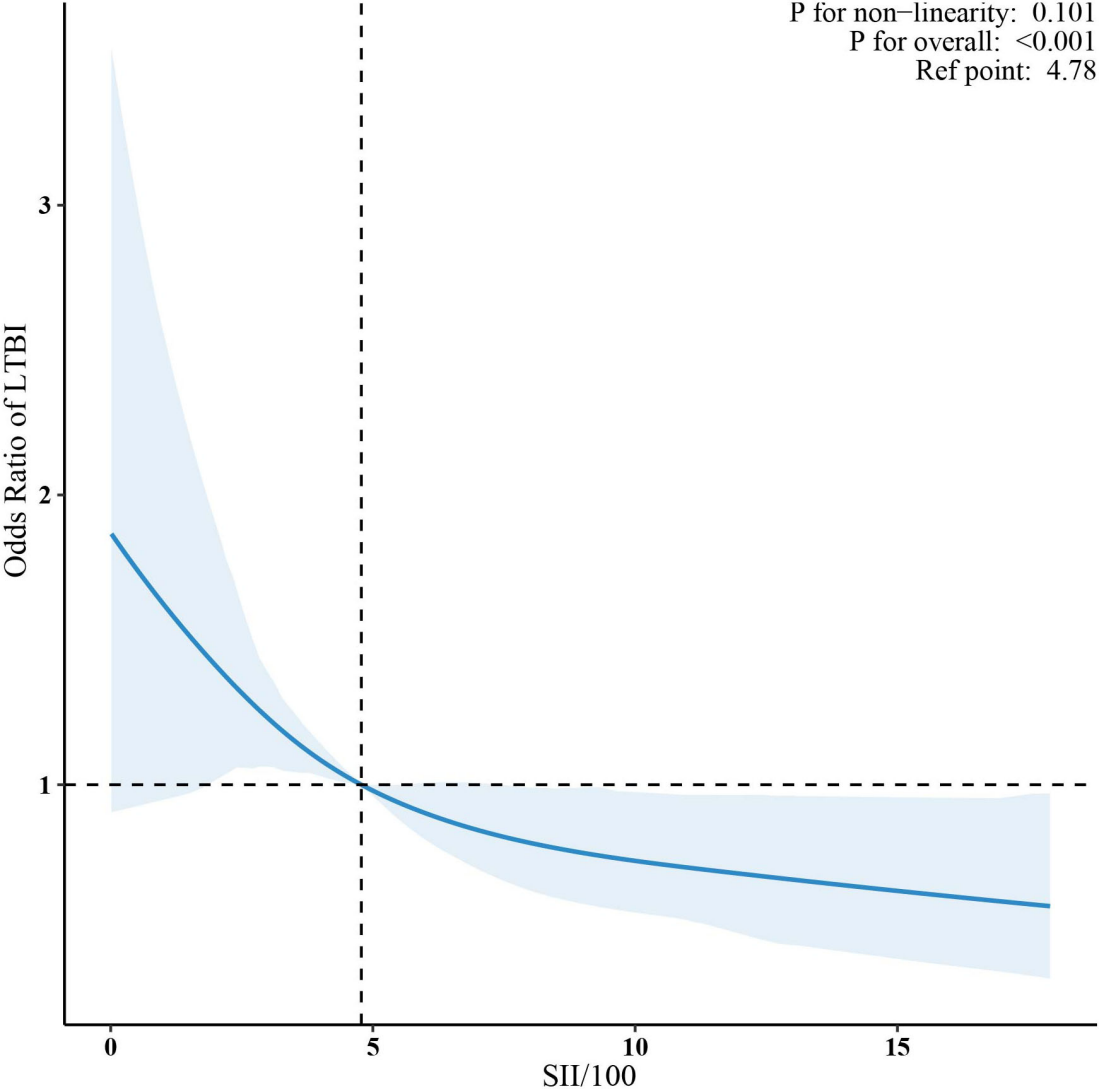
Association between SII/100 and LTBI. The solid red lines represent the predicted odds ratios, while the dashed red lines indicate the 95% confidence intervals. Adjusted for sex, age, race/ethnicity, family income, education level, marital status, body mass index, diabetes, coronary heart disease, hypertension, smoking status, alcohol use, HIV, HBV, HCV. Only the top 99% of SII data are shown. SII, systemic immune-inflammation index; LTBI, latent tuberculosis infection.

Sample-weighted multivariable logistic regression analysis indicated a consistent negative association between SII and LTBI across all models (Table 2). A 100-unit increase in SII was associated with a 6% reduction in the odds of LTBI, both in unadjusted (OR = 0.92, 95% CI: 0.90–0.95) and fully adjusted models (OR = 0.94, 95% CI: 0.90–0.97). When analyzed by quartiles, participants in the highest SII quartile (Quartile 4) had a 42% lower likelihood of LTBI compared to those in Quartile 1 (OR = 0.58, 95% CI: 0.41–0.81) in the fully adjusted model. The trend test indicated a statistically significant trend (*p* < 0.05). The linear relationship between SII and LTBI was confirmed by weighted RCS (*p* for non-linearity >0.05) (Figure 2).

**TABLE 2:** Associations between SII/100 and LTBI.

Variable	n.event_%	Model I		Model II		Model III	
		OR (95%CI)	*P*-Value	OR (95%CI)	*P*-Value	OR (95%CI)	*P*-Value
SII/100	1068 (11.3)	0.92 (0.90∼0.95)	<0.001	0.94 (0.91∼0.97)	<0.001	0.94 (0.90∼0.97)	0.002
**SII/100 quartiles**							
Quartile 1	346 (14.6)	1 (Ref)		1 (Ref)		1 (Ref)	
Quartile 2	269 (11.3)	0.64 (0.51∼0.81)	<0.001	0.74 (0.57∼0.97)	0.030	0.74 (0.56∼0.99)	0.045
Quartile 3	251 (10.6)	0.57 (0.46∼0.71)	<0.001	0.64 (0.51∼0.82)	0.001	0.63 (0.48∼0.83)	0.005
Quartile 4	202 (8.5)	0.51 (0.39∼0.66)	<0.001	0.60 (0.45∼0.80)	0.002	0.58 (0.41∼0.81)	0.006
P for trend			<0.001		<0.001		0.003

Model I: unadjusted; Model II: adjusted for: sex, age, race/ethnicity, family income, education level, marital status; Model III: adjusted for: sex, age, race/ethnicity, family income, education level, marital status, body mass index, diabetes, coronary heart disease, hypertension, smoking status, alcohol use, HIV, HBV, HCV. SII, systemic immune-inflammation index; LTBI, latent tuberculosis infection; OR, odds ratio; CI, confidence interval; Ref, reference; HIV, human immunodeficiency virus; HBV, hepatitis B virus; HCV, hepatitis C virus.

### 3.3 Subgroup analyses

**FIGURE 3:**
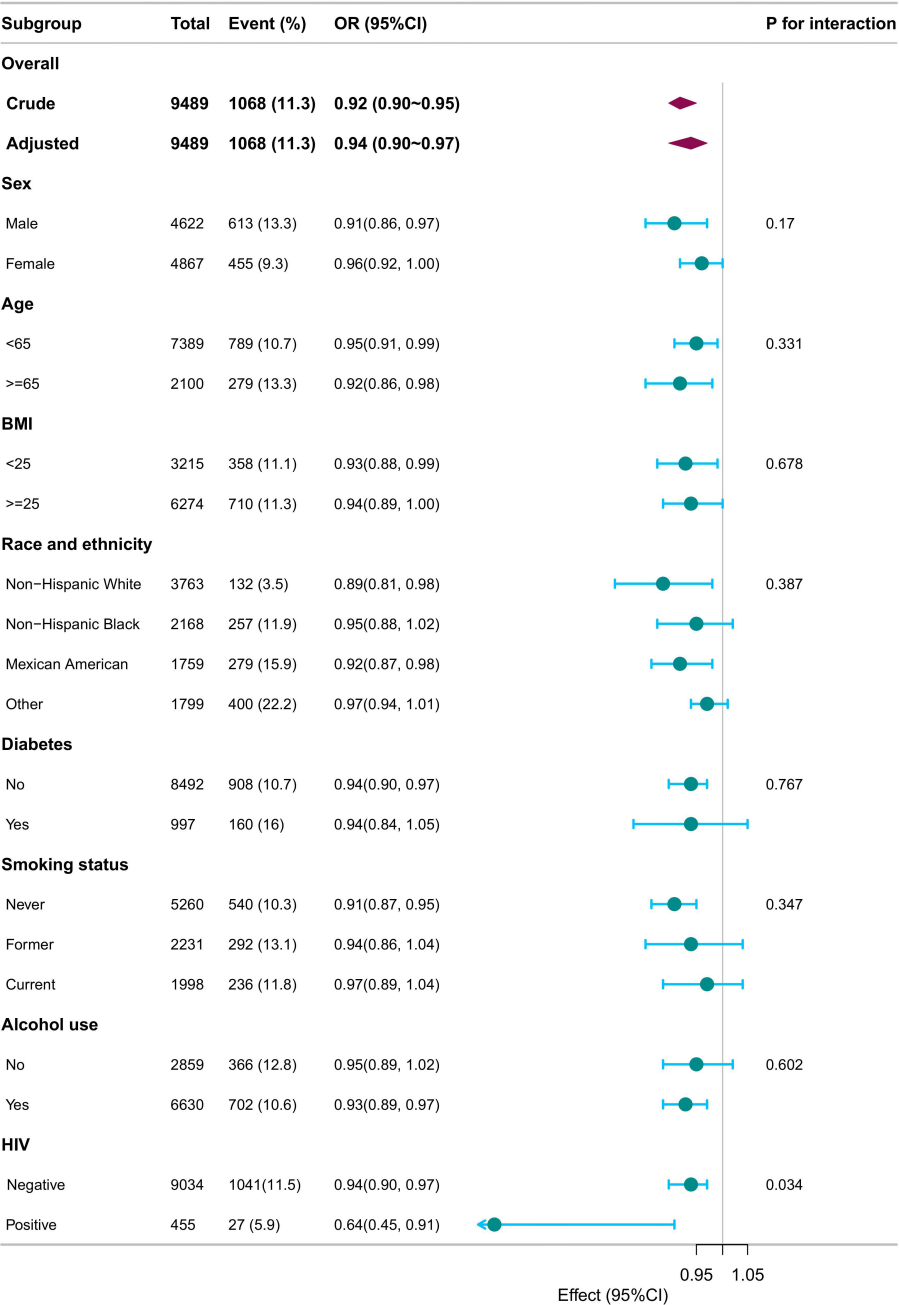
Subgroup analysis of SII/100 and LTBI. Adjusted for sex, age, race/ethnicity, family income, education level, marital status, body mass index, diabetes, coronary heart disease, hypertension, smoking status, alcohol use, HIV, HBV, HCV except for the stratification component itself. SII, systemic immune-inflammation index; LTBI, latent tuberculosis infection.

Subgroup analyses were performed based on sex, age (< 18 years, ≥ 18 years), BMI (< 25 kg/m^2^, ≥ 25 kg/m^2^), race/ethnicity, diabetes, smoking status, alcohol use, and HIV status. Adjustments were made for sex, age, race/ethnicity, family income, education level, marital status, body mass index, diabetes, coronary heart disease, hypertension, smoking status, alcohol use, HIV, HBV, and HCV. The associations remained consistent across all subgroups, with no significant interactions observed (p for interaction >0.05), except for HIV status (p for interaction = 0.034) (Figure 3).

## 4 Discussion

This study is the first to demonstrate a significant inverse association between SII and LTBI in a United States adult population sample from NHANES.

The systemic immune-inflammation index (SII), a novel composite biomarker derived from platelet, neutrophil, and lymphocyte counts, has become a validated indicator of systemic inflammation and immune dysregulation (17). Changes in peripheral blood cell profiles, reflected by SII, provide a reliable measure of chronic low-grade inflammation. Numerous studies have highlighted its clinical significance across various diseases, including oncology (24), cardiovascular conditions (25), and metabolic syndrome (26). SII has also been associated with infectious diseases. Wang et al. found that individuals with HIV had notably lower SII levels compared to those without the infection (27). A multicenter study further showed that higher SII is an independent predictor for intensive care unit (ICU) admission in COVID-19 patients (28). Additionally, elevated SII has been linked to increased mortality in Clostridium difficile infections (29).

Recent studies have increasingly focused on the relationship between the SII and tuberculosis. Yu et al. observed that SII levels were notably higher in patients with active pulmonary tuberculosis compared to those with non-tuberculous lung diseases (12). Kerget et al. also reported that SII levels were considerably higher in patients with tuberculous lymphadenitis than in those with granulomatous disease and reactive lymphadenopathy (30). Furthermore, Liu et al. showed a significant link between SII levels and depression or anxiety symptoms in tuberculosis patients (31). Huang et al. further found that SII levels were markedly higher in patients who experienced restenosis after interventional treatment for tuberculous airway stenosis, compared to those who did not (32). These results imply that SII, as a comprehensive marker of systemic inflammation and immune function, may offer valuable insights in the context of tuberculosis.

However, large-scale studies exploring the link between SII and LTBI remain scarce. Our research identifies a significant negative association between SII and LTBI, with higher SII values corresponding to a reduced risk of LTBI. This contrasts with the inflammatory profile of active TB, where elevated SII reflects ongoing immune activation (12). However, LTBI represents a specific stage within the continuum from tuberculosis infection to disease, characterized by a dynamic state of immune equilibrium in which Mtb is contained without apparent clinical manifestations (33). Interestingly, prior studies have reported inverse associations between LTBI and inflammatory markers such as the monocyte-to-lymphocyte ratio (MLR) and neutrophil-to-lymphocyte ratio (NLR) (18, 34). In addition, the SII has been shown to be negatively correlated with HIV status (27). Consistently, our subgroup analysis revealed a statistically significant interaction between SII and HIV status, with the inverse association between SII and LTBI being more pronounced among individuals living with HIV.

The systemic immune-inflammation index (SII) combines platelet, neutrophil, and lymphocyte counts, all of which are critical in the immune response to tuberculosis (35, 36). Neutrophils play a vital role in both innate and adaptive immunity, crucial for preventing tuberculosis infection (37). They are phagocytic cells within early TB granulomas, responsible for killing Mtb through oxidative damage (38). A study on active pulmonary tuberculosis found that neutrophils were the predominant cell type infected by Mtb in respiratory secretions (39). A study in the United Kingdom showed that the risk of tuberculosis infection among contacts of pulmonary tuberculosis patients was independently and negatively correlated with peripheral blood neutrophil counts. Neutrophil depletion reduced whole blood’s ability to inhibit the growth of Mycobacterium bovis BCG and Mtb by 7.3-fold and 3.1-fold, respectively (40). Recent research has emphasized the complex role of platelets in tuberculosis pathogenesis. Platelets participate in the immune response against Mtb through the production of host defense peptides and cytokines (41). Platelets can also release a variety of cytokines and chemokines, such as PF4 and VEGF-A, which play a key role in promoting chemotaxis and activation of monocytes and enhancing inflammatory responses (42). However, platelets may also adversely affect primary progressive tuberculosis by limiting the production of reactive oxygen species in lung-resident myeloid cells, thereby hampering antimicrobial defense (43). Thus, elevated SII values likely reflect heightened neutrophil and platelet activity, contributing to the control of Mtb growth and spread.

These findings suggest that SII may offer useful insights into host immune responses to Mtb infection. Based on routinely available hematological parameters, SII holds potential as a supportive tool for LTBI risk stratification, particularly in resource-limited settings. However, its application should be interpreted cautiously and within the context of broader clinical and epidemiological assessment.

This study utilizes data from the NHANES database and adopts a cross-sectional design to explore the relationship between SII and LTBI. Nonetheless, several limitations must be acknowledged. Firstly, the cross-sectional nature of the study prevents the establishment of causality or the identification of temporal sequence. Thus, it remains uncertain whether elevated SII levels precede LTBI development, result from LTBI, or reflect shared underlying risk factors. Future longitudinal studies are warranted to clarify these relationships. Secondly, some potential confounding factors, such as chronic illnesses, medication usage, BCG vaccination, and immunosuppressive therapy, are inadequately recorded in the NHANES dataset, which may influence the accuracy of the results. Thirdly, although LTBI was defined as a positive result on either QFT-GIT or TST, both tests have known limitations. QFT-GIT may yield false-negative results, particularly in immunocompromised or elderly individuals, whereas TST may produce false-positive results due to prior BCG vaccination or exposure to non-tuberculous mycobacteria. Finally, SII was assessed at a single time point, which may not reflect its temporal variability. Moreover, as a non-specific inflammatory marker, SII can be elevated due to various conditions such as infections, cancers, and autoimmune diseases, potentially leading to misclassification or residual confounding. Future studies should implement a longitudinal approach, consider additional confounders, and replicate the results in different population groups.

## 5 Conclusion

Our findings demonstrate a significant inverse association between SII and LTBI, suggesting that SII may assist in LTBI risk stratification among adults. However, given its non-specific nature, further longitudinal studies are needed to validate these findings and clarify the potential clinical and public health implications of SII in LTBI screening and management.

## Data Availability

The original contributions presented in this study are included in this article/supplementary material, further inquiries can be directed to the corresponding author.

## Footnotes

1
http://www.R-project.org
